# miR-410-3P inhibits adipocyte differentiation by targeting IRS-1 in cancer-associated cachexia patients

**DOI:** 10.1186/s12944-021-01530-9

**Published:** 2021-09-25

**Authors:** Diya Sun, Zuoyou Ding, Lei Shen, Fan Yang, Jun Han, Guohao Wu

**Affiliations:** 1 Department of General Surgery, Zhongshan Hospital of Fudan University, Shanghai, China; 2 Shanghai Clinical Nutrition Research Centre, Shanghai, China

**Keywords:** Cancer-associated cachexia, microRNA, Adipose tissue loss, miR-410-3p, 3′-untranslated regions, Insulin Receptor Substrate 1, Adipose differentiation, Exosomes

## Abstract

**Backgrounds:**

Cancer-associated cachexia (CAC) is a metabolic syndrome characterized by progressive depletion of adipose and muscle tissue that cannot be corrected by conventional nutritional therapy. Adipose tissue, an important form of energy storage, exhibits marked loss in the early stages of CAC, which affects quality of life and efficacy of chemotherapy. MicroRNAs (miRNAs) are a class of noncoding RNAs that widely exist in all kinds of eukaryotic cells and play regulatory roles in various biological processes. However, the role of miRNAs in adipose metabolism in CAC has rarely been reported. This study attempted to identify important miRNAs in adipose metabolism in CAC and explore their mechanism to identify a new predictive marker or therapeutic target for CAC-related adipose tissue loss (CAL).

**Methods:**

In this study, miRNA sequencing was firstly used to identify differentially expressed miRNAs related to CAL and the reliability of the conclusions was verified in large population samples. Furthermore, functional experiments were performed by up and down regulating miR-410-3p in adipocytes. The binding of miR-410-3p to Insulin Receptor Substrate 1 (IRS-1) was verified by Luciferase reporter assay and functional experiments of IRS-1 were performed in adipocytes. Finally, the expression of miR-410-3p in serum exosomes was detected.

**Results:**

miR-410-3p was selected as differentially expressed miRNA through screening and validation. Adipogenesis was suppressed in miR-410-3p upregulation experiment and increased in downregulation experiment. Luciferase reporter assay showed that miR-410-3p binds to 3′ non-coding region of IRS-1 and represses its expression and ultimately inhibits adipogenesis. miR-410-3p was highly expressed in serum exosomes of CAC patients, which was consistent with results in adipose tissue.

**Conclusions:**

The expression of miR-410-3p was higher in subcutaneous adipose tissues and serum exosomes of CAC patients, which significantly inhibits adipogenesis and lipid accumulation. The study shows that miR-410-3p could downregulate IRS-1 and downstream adipose differentiation factors including C/EBP-a and PPAR-γ by targeting 3′ noncoding region.

**Supplementary Information:**

The online version contains supplementary material available at 10.1186/s12944-021-01530-9.

## Introduction

Cancer-associated cachexia (CAC) is a metabolic syndrome characterized by progressive muscle and adipose tissue loss that is reportedly responsible for the deaths of many cancer patients [1]. CAC negatively affects patient quality of life and the efficacy of chemotherapy [2, 3]. Although skeletal muscle loss has been emphasized in definition, previous studies have shown that most CAC patients also experience massive adipose tissue wasting. CAC-related adipose tissue loss (CAL) has been identified as an important marker of poor prognosis [4, 5]. This phenomenon often appears earlier than skeletal muscle wasting [5], and such a comprehensive study of CAL would not only be valuable for the early diagnosis and treatment of CAC but also as a potential marker for patient prognosis.

In mammals, adipose tissue is primarily divided into white adipose tissue (WAT) and brown adipose tissue (BAT). Among these two classifications, BAT is minimally distributed in adults, while WAT primarily makes up subcutaneous and visceral adipose tissue, playing distinctive roles in metabolic regulation [6]. Numerous findings have shown evidence of dysfunctional adipogenesis, insufficient lipid accumulation, adipokine parasecretion and vigorous adipose consumption in every type of adipose tissue in CAC patients [7–9], which collectively results in CAL. Furthermore, BAT maintains body temperature through adaptive thermogenesis, a process that involves uncoupling mitochondrial respiration that is primarily mediated by uncoupling protein 1 (UCP1) [10]. This consumption of adipose tissue is similar to the browning phenomenon observed in the WAT of CAC patients, indicating the need for additional studies in this area concerning CAC [11].

MicroRNAs are short-chain RNA molecules consisting of approximately 19–24 bp nucleotides [12]. Their role has been confirmed in the regulation of gene expression by targeted binding to hundreds of mRNAs, which ultimately influences numerous biological processes [13, 14]. Research shows that more than 60 % of human protein-coding gene mRNAs constitute miRNA binding sites in 3′-untranslated regions (3′-UTRs) and that the dysregulation of miRNAs may severely impact normal biological processes *in vivo*, ultimately leading to disease [15]. Various studies have shown that differential miRNA expression may play a role in tumorigenesis, invasion and metastasis in several cancers. This is also evident in nonneoplastic diseases, such as Alzheimer’s disease [16–18]. Therefore, identifying differentially expressed miRNAs in a complex miRNA expression database, predicting target mRNAs, and searching for possible biological roles is a crucial method for understanding the occurrence and development of disease.

IRS-1 is the most common insulin receptor substrate. Previous studies have found that insulin performs important regulatory function in adipose differentiation and IRS-1 could affect differentiation factors including PPAR-γ and C/EBP-a and thus regulates adipose differentiation [19].

Exosomes are vesicles 50–150 nm in diameter that are secreted into the external environment by most cells [20]. These vesicles are composed of lipid bilayers and contain various substances, such as proteins, glycans, lipids, RNAs, DNAs and small miRNA molecules [20, 21]. These substances are taken up by recipient cells via endocytosis and affect their biological processes [22]. Thus, exosomes are regarded as important cellular mediators of communication. In short, miRNAs can be secreted into body fluids with the help of exosomes to regulate the biological functions of targeted cells from a distance.

Due to the lack of research concerning miRNAs in CAL, this study aimed to discover novel differentially expressed miRNAs involved in the regulation of subcutaneous adipose tissues in CAC patients via a rigorous screening process. Current study identified miR-410-3p to have an inhibitory function in adipogenesis with upregulation and downregulation experiments that determined its role in the inhibition of adipogenic differentiation by binding to the 3′-UTR of IRS-1 mRNA. Finally, this study got a surprising discovery that miR-410-3p was present at extremely high levels in exosomes by examining serum exosomes from CAC patients. This elevated expression from patient adipocytes may represent a novel finding indicating that tumours use this mechanism to remotely regulate nutrient metabolism.

## Methods

### Patients and data collection

Patients from the General Surgery Department of Zhongshan Hospital, Fudan University were recruited from June 2019 to September 2020 to participate in this study. All patients written informed consent. The inclusion criteria were as follows: (1) Patients with histological diagnosis of gastric cancer; (2) Patients required radical surgery in hospital. The exclusion criteria were as follows: (1) Patients who had received radiotherapy, chemotherapy or other medications before surgery; (2) Patients with severe cardiovascular or cerebrovascular diseases, liver or kidney insufficiency, diabetes or HIV/AIDS. This study followed the standards based on the 2011 international consensus about cachexia: (1) more than 5 % weight loss within six months; (2) BMI < 20 and more than 2 % weight loss within six months. The patients were divided into cachexia group and non-cachexia group, and basic information such as height, weight, gender, age, TNM staging were obtained from the medical records, as well as the preoperative blood routine examination of inflammation and nutritional indicators.

### Cell culture and differentiation

The white preadipocytes provided by Dr. Qiurong Ding, Shanghai Institute of Nutrition and Health, Chinese Academy of Sciences [23], Cultured in a primary medium composed of high glucose DMEM, 20 % FBS and 1 % penicillin / Streptomycin at 37 °C in 5 % CO2. The differentiation induction medium was prepared as follows: 10 % FBS + DMEM, 5 µg/mL insulin, 1000nM dexamethasone, 0.5mM isobutyl-1-methylxanthine (IBMX) and 1µM rosiglitazone. It was replaced with maintenance medium (5 µg/mL insulin) and maintained for 4 days after 2 days of differentiation.

### miRNA sequencing

miRNA sequencing (miRNA-seq) was performed by OE Biotech Co., Ltd. (Shanghai, China). There were 6 samples in this project. CleanReads of each sample ranged from 20.91 to 29.58 M, the genome contrast ratio was 93.4-95.56 %, and the known miRNA contrast ratio was 44.42–50.82 %. The total number of known miRNAs in all samples was 1634, and the total number of newly predicted miRNAs was 836. Relevant data had been uploaded to GEO(GSE174128).

### RNA analysis

TRIzol was used to extract total RNA. The first cDNA chains of miRNA were produced by reverse transcription (Tailing Reaction), and the transcriptome cDNAs were synthesized by oligo (DT) priming and M-MLV reverse transcriptase. qPCR analyses were performed on a QuantStudio 6 fluorometer using SYBR® green chimeric fluorometry. Primers for PCR are listed in Supporting Information Table S1.

### Protein analysis

Protein lysates were stored in 1× SDS and separated by SDS-PAGE. The nitrocellulose membranes transferred with proteins were blocked in 5 % BSA, incubated overnight at 4 °C with specific primary antibodies, washed and incubated for 1 h with the corresponding secondary antibodies. A Tanon 5200 automated chemiluminescence imaging analysis system was subsequently implemented. The following primary antibodies were used in this study: tubulin (2146 S; Cell Signaling Technology, Danvers, USA; 1:1000), C/EBP-α (2295 S; Cell Signaling Technology, Danvers, USA; 1:1000), PPAR-γ (2435T; Cell Signaling Technology, Danvers, USA; 1:1000), C/EBP-β (3082 S; Cell Signaling Technology, Danvers, USA; 1:1000), LPL (sc-373; Santa Cruz Biotechnology, Dallas, USA; 1:800), FABP4 (2120 S; Cell Signaling Technology, Danvers, USA; 1:1000), and IRS-1 (ab52167; Abcam, Cambridge, UK; 1:1000).

### RNA transfection

Lipofectamine™ RNAiMAX transfection reagent (Invitrogen) was used to transfect 100 nM of small interfering RNAs, miRNA mimics and miRNA inhibitors (concentrations see below) when cell density was approximately 70 % according to the manufacturer’s instructions. Normal medium as described above was used after 8 h of transfection to achieve complete growth and differentiation. The siRNA oligos, miRNA mimics and miRNA inhibitors provided by Gene Pharma (Shanghai, China) and the sequences used are listed in Supporting Information Tables S2 and S3.

### Oil red O staining

White adipocytes were fixed in 4 % paraformaldehyde for 30 min at room temperature, stained for 1 h using 0.3 % Oil red O solution and washed three times. Isopropanol was used to wash the stained cells to quantitatively assess lipid accumulation, and the absorbance was determined at 520 nm.

### Free fatty acid analysis

The free fatty acid content of liquid samples was measured using the enzyme labelling method, and the cell culture supernatant was directly used in this experiment. A free fatty acid assay kit (Nanjing Jiancheng Bioengineering Institute) was used according to the manufacturer’s instructions.

### Luciferase reporter assay

For dual luciferase analysis, 1.2 × 104 HEK293T cells in 96 well plates and 100 ng luciferase reporter plasmid were transiently cotransfected with wild-type (WT) or mutant (MT) mir-410-3p expressing Renilla luciferase (Promega) using Lipofectamine 2000 reagent. After 24 h of transfection, the cells were harvested. Using dual luciferase ® The reporter analysis system (Promega Corporation) measured the activities of firefly and Renilla luciferase. Firefly luciferase values were normalized to Renilla signals and the ratio of firefly / Renilla values was determined. All experiments were conducted in triplicate.

### Extraction of exosomes and exosomal miRNAs

A special rapid exosome extraction kit (EZBioscience) was used to extract exosomes from serum. Then, 500 µL serum was mixed with 125 µL exosome precision reagent (EPR). After resting overnight at 4 °C and centrifuging at 1000×g for 30 min, the supernatant was discarded, and the pellet was resuspended in 1×PBS and transferred to the purification column. The purified exosomes were collected after centrifugation at 4 °C and 4000×g for 5 min. The preparation and analysis of exosomal miRNAs were the same as described above.

### Transmission electronic microscopy

The obtained exosomes were placed onto copper mesh and stained with 1 % uranyl acetate for 3 min. After drying, the samples were observed at 80.0 kV on a Hitachi ht7700 transmission electronic microscope (TEM) according to the manufacturer’s instructions.

### Nanoparticle tracking analysis

Nanoparticle tracking analysis was used to quantify the number and size of exosomes present in the serum. Exosomes stored in PBS were diluted 100- to 500-fold to make approximately 100 objects per frame. The NanoSight system was used for complete analysis.

### Statistical analysis

The data were analysed using unpaired two-tailed Student’s t-tests. All data are presented as the mean ± SEM. The chi-square test was also used to analyse the correlation between miRNAs and clinical indicators. *P* < 0.05 was considered statistically significant.

## Results

### Identification of differentially expressed miRNAs in cachexia and noncachexia cancer patients

This study first chose three gastric cancer patients with cachexia. To obtain significant results, these three patients were enrolled based on remarkable weight loss, with an average of 14.63 % (Supporting Information Table S4), which far exceeded the international diagnostic standard for cachexia (5 % weight loss). Subsequently, three gastric cancer patients without significant weight change within half a year were selected as the control group while ensuring that their general characteristics were similar, including sex and age.

**Fig. 1:**
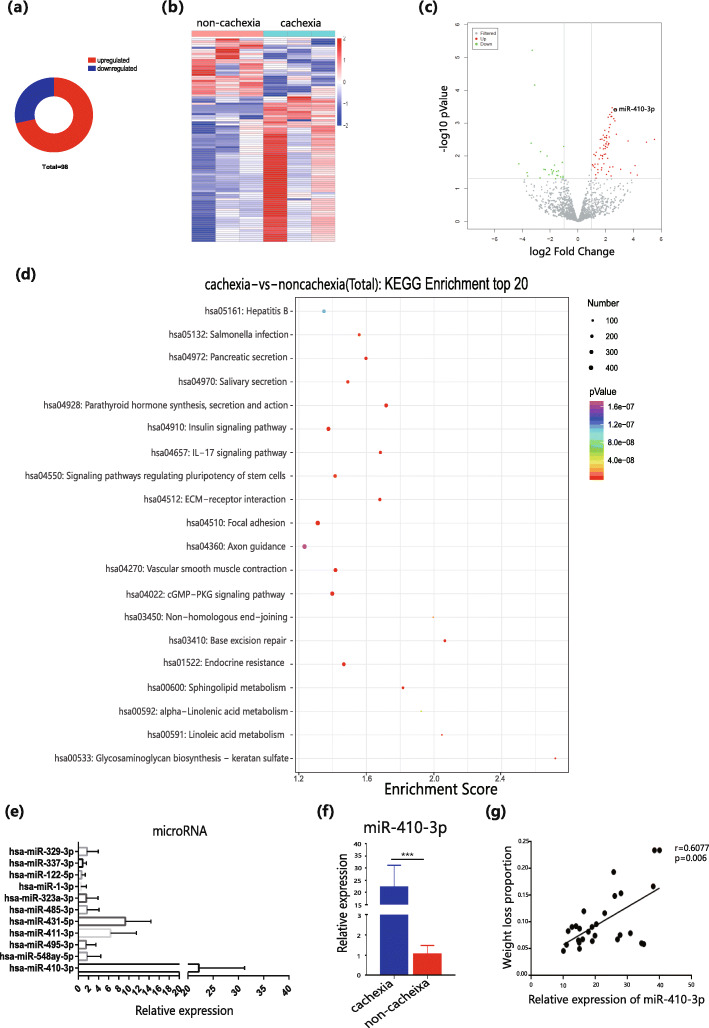
Identifying differentially expressed miRNA in cachexia and non-cachexia cancer patients. **a**. The differentially expressed miRNA numbers in miRNA sequencing. **b** and **c**. The heatmap and volcano plots of the differentially expressed miRNAs in miRNA sequencing. **d**. Enrichment analysis top 20 in KEGG database (sorted by - log_10_*P* value of each entry) **e**. qPCR analyses of 11 differential miRNAs with minimum *P-*value in CAC groups. (non-cachexia groups’ relative expression was 1). **f** and **g**. qPCR analyses of miR-410-3p in CAC and non-cachexia groups and the correlation analysis of miR-410-3p expression and proportion of weight loss in CAC patients. All results are expressed as mean ± SEM. * *P* < 0.05, ** *P* < 0.01, *** *P* < 0.001

A small piece of subcutaneous adipose tissue was resected from the abdomen of these six patients at the time of surgery. These tissues were used in miRNA sequencing and yielded 98 differential miRNAs, including 70 upregulated and 28 downregulated miRNAs (Fig. 1a). The heatmap and volcano plots of the differentially expressed miRNAs are shown in Fig. 1b and c. This study proceeded with the target gene enrichment analysis of differential miRNAs in the GO (Supporting Information Figure S1) and KEGG (Fig. 1d) databases. The most remarkable result in the KEGG database functional enrichment analysis was the high abundance of target genes in the insulin signalling pathway, which plays an important role in adipocyte differentiation and could provide assistance for selecting miRNA target genes and related mechanisms.

To confirm the reliability of the miRNA sequencing results and select the miRNA with the highest influence on adipose metabolism in cachexia, additional 60 gastric cancer patients were selected, 30 of whom were patients with cachexia and 30 of whom were without cachexia. A piece of subcutaneous adipose tissue was resected at the abdominal incision as previously described. Ultimately 11 differential miRNAs were selected out of 98 that had a minimum *P-*value and│log2FC│> 4 (Supporting Information Table S5). From the results of 11 miRNAs in 50 subcutaneous adipose samples by qPCR (Fig. 1e), miR-410-3p were selected for subsequent experiments, as it had the largest fold change and *P-*value < 0.001 (Fig. 1f), which was highly consistent with the sequencing result. To learn more about the function of miRNAs in adipocyte metabolism in CAC patients, this study explored the correlation between miR-410-3p and the proportion of weight loss in CAC patients. The results demonstrated a strong correlation between these two variables, with an r value of 0.6 and a *P*-value of 0.006 (Fig. 1 g).

Finally, this study explored the correlation between the expression of miR-410-3p and clinical indicators in these cancer patients. The results revealed higher expression in male patients and its relevance to TNM staging and serum albumin in cancer patients. Most importantly, high expression of miR-410-3p was significantly correlated with weight loss, indicating its close relationship with CAC (Table 1). Notably, miR-410-3p was related to IL-6 because of its strong association with CAL observed in previous reports [24].

**Table 1:** Clinical correlation of miR-410-3p expression in cancer patients

	miR-410-3p			
Features	Low (*n* = 30)	High^a^ (*n* = 30)	t/χ2	*P*
Gender			4.8	0.028
Male	16	24		
Female	14	6		
Age (years)			0.606	0.436
> 65	12	15		
≤ 65	18	15		
Tumor stage			8.531	0.003
I + II	17	6		
III + IV	13	24		
Weight (kg)	62.3 ± 8.06	59.73 ± 11.6	0.834	0.410
BMI (kg/m2)	22.6 ± 2.55	21.71 ± 2.2	1.144	0.260
Body weight loss (%)	0.23 ± 0.09	9.76 ± 4.12	11.862	< 0.001
IL-6 (pg/mL)	3.81 ± 1.78	6.96 ± 2.74	2.385	0.024
TNF-a (pg/mL)	9.57 ± 5.98	12.45 ± 7.83	1.164	0.253
TP (g/L)	65.21 ± 5.44	64.27 ± 4.77	0.57	0.574
ALB (g/L)	41.57 ± 4.41	39.1 ± 3.92	1.947	0.044

^a^According to the median expression level of miR-410-3p in all patients, the expression with lower than 50th percentile was classified as low expression group; the patients with higher than 50th percentile was classified as high expression group

### miR-410-3P inhibits adipogenesis *in vitro*

**Fig. 2:**
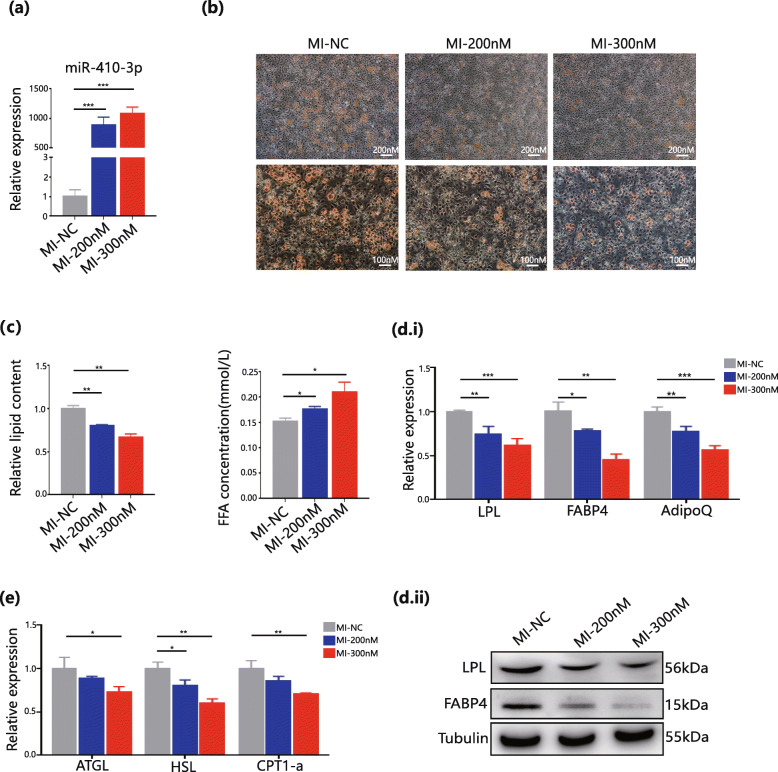
Upregulation of miR-410-3P inhibited adipogenesis. **a**. qPCR analyses of miR-410-3p in normal control (NC) group and miR-410-3p mimics concentration gradient (200nM or 300nM) group. **b**. Oil Red O staining observed with 10X and 20X eyepiece in NC group and miR-410-3p mimics concentration gradient group. **c**. quantitative analysis of lipid accumulation and free fatty acids of cell supernatant in NC group and miR-410-3p mimics concentration gradient group. **d.i**. qPCR analyses of adipogenic markers in NC group and miR-410-3p mimics concentration gradient group. **d.ii**. Western Blot analyses of adipogenic markers in NC group and miR-410-3p mimics concentration gradient group. **e**. qPCR analyses of lipolysis markers in NC group and miR-410-3p mimics concentration gradient group. All results are expressed as mean ± SEM. * *P* < 0.05, ** *P* < 0.01, *** *P* < 0.001

To analyse the role of miR-410-3p in adipocyte metabolism, miR-410-3p mimics and inhibitors were transfected into white preadipocytes using a concentration gradient, followed by induction of differentiation. There was significant upregulation of miR-410-3p expression in the mimic group on day 6 of differentiation (Fig. 2a), which inhibited adipogenesis and lipid accumulation, as shown by the decrease in oil red staining (Fig. 2b). In addition, a more powerful inhibitory effect was observed at higher concentrations of miR-410-3p. The quantitative analysis of lipid accumulation and free fatty acids in the cell supernatant also supported this finding, which was indicated by low levels of lipid accumulation and a high concentration of free fatty acids in the cell supernatant in the miR-410-3p mimic group (Fig. 2c). As hypothesized, the qPCR results showed that adipogenic markers, such as lipoprotein lipase (LPL), fatty acid binding protein 4 (FABP4) and adiponectin (AdipoQ), were significantly downregulated in the mimic group, which was consistent with the western blot results of LPL and FABP4 (Fig. 2d). This study also examined key genes for lipolysis in CAL, such as ATGL, HSL, and CPT1-a [25], and the qPCR results similarly suggested low expression in the miR-410-3p mimic group, which is shown in Fig. 2e. This conclusion illustrated that there is a regulatory role for miR-410-3p in adipogenesis and that the simultaneous downregulation of lipolysis should not be influenced by miR-410-3p but is more likely a feedback effect on adipogenesis. Lipolysis in CAC patients was enhanced; however, the overexpression experiment with miR-410-3p did not support this observation.

**Fig. 3:**
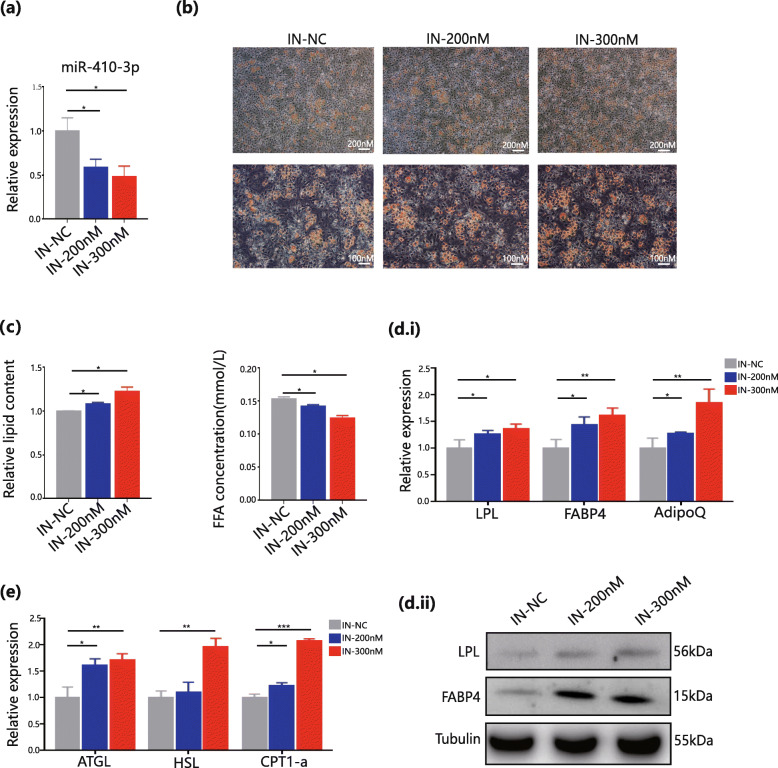
Downregulation of miR-410-3P enhanced adipogenesis. **a**. qPCR analyses of miR-410-3p in NC group and miR-410-3p inhibitors concentration gradient group. **b**. Oil Red O staining observed with 10X and 20X eyepiece in NC group and miR-410-3p inhibitors concentration gradient (200nM and 300nM) group. **c**. quantitative analysis of lipid accumulation and free fatty acids of cell supernatant in NC group and miR-410-3p inhibitors concentration gradient group. **d.i**. qPCR analyses of adipogenic markers in NC group and miR-410-3p inhibitors concentration gradient group. **d.ii**. Western Blot analyses of adipogenic markers in NC group and miR-410-3p inhibitors concentration gradient group. **e**. qPCR analyses of lipolysis markers in NC group and miR-410-3p inhibitors concentration gradient group. All results are expressed as mean ± SEM. * *P* < 0.05, ** *P* < 0.01, *** *P* < 0.001

In the inhibitor group, the expression of miR-410-3p was downregulated by inhibitors in a concentration-dependent manner on day 6 of differentiation, with 50 % knockdown at the highest concentration (Fig. 3a). These results suggest a significant increase in oil red staining and a low concentration of free fatty acid cell supernatant in the miR-410-3p inhibitor group. However, the enhancement in adipogenesis and lipid accumulation was not strong compared to that in the mimic group (Fig. 3b and c). Simultaneously, the LPL and FABP4 genes and proteins were also upregulated in the inhibitor group, which confirmed this finding (Fig. 3d). Next, this study examined lipolysis enzymes, such as ATGL, HSL, and CPT1-a, and observed upregulation in the miR-410-3p inhibitor group (Fig. 3e). Therefore, *in vitro* experiments regarding the upregulation and downregulation of miR-410-3P confirmed the role of miR-410-3P in adipogenesis inhibition.

### IRS-1 is a target of miR-410-3P

**Fig. 4:**
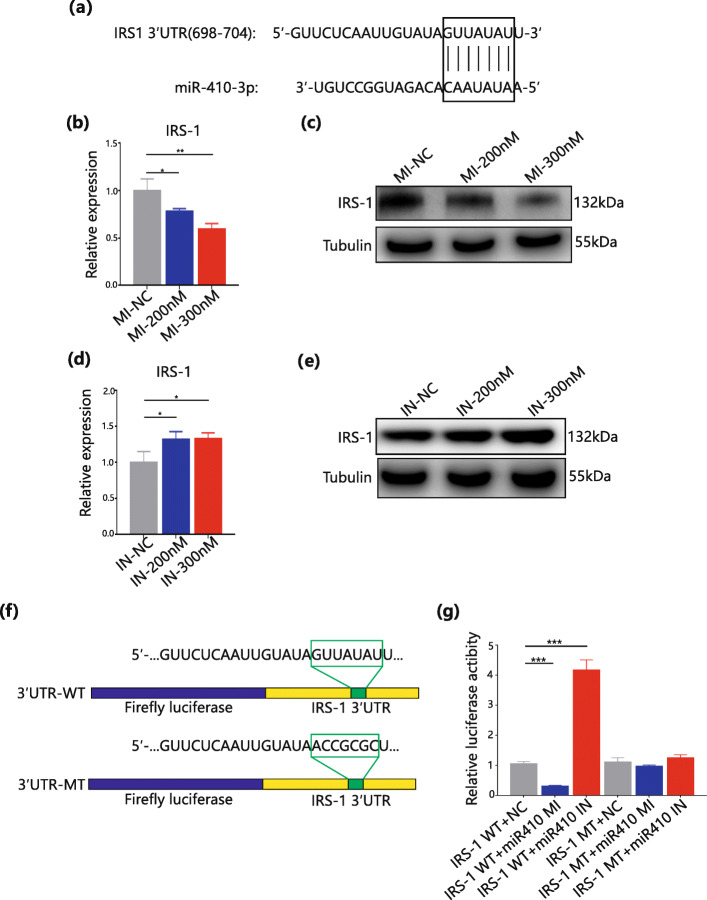
IRS1 is a target of miR-410-3P. **a**. The matching of miR-410-3p with 3’-untranslated regions (UTR) of IRS-1. **b** and **c**. qPCR analyses and Western Blot analyses of IRS-1 in NC group and miR-410-3p mimics concentration gradient (200nM and 300nM) group. **d** and **e**. qPCR analyses and Western Blot analyses of IRS-1 in NC group and miR-410-3p inhibitors concentration gradient (200nM and 300nM) group. **f**. the different sequences of luciferase reporter plasmid weird type (WT) and mutated type (MT).**g**. Luciferase reporter assay in HEK293T cells. All data are expressed as mean ± SEM. * *P *< 0.05, ** *P *< 0.01, *** *P* < 0.001

Next, this study investigated a possible target gene for miR-410-3p to uncover the mechanisms of how miR-410-3p inhibits adipogenesis. With the help of miRNA target databases such as TargetScan, IRS-1 was selected as a potential target because miR-410-3p is believed to downregulate IRS-1 expression by binding to its 3′-UTR (Fig. 4a). Other targets mRNAs of miR-410-3P were shown in Supporting Information Table S6. Based on previous experiments, IRS-1 exhibited corresponding changes in both gene and protein levels in response to the regulation of miR-410-3p (Fig. 4b-e). In contrast, there was an opposite relationship between miR-410-3p and IRS-1 mRNA. Based on the TargetScan database, a miR-410-3p targeting site was predicted in the 3′-UTR of IRS-1 and then designed wild type (WT) and mutated luciferase reporter plasmids connected to the 3′-UTR of IRS-1 (Fig. 4f). A luciferase reporter assay showed that luciferase activity was dependent on the 3′-UTR of IRS-1, which was significantly downregulated in response to high concentrations of miR-410-3p and significantly upregulated at low concentrations. This was not seen in a replicated experiment using MT plasmids (Fig. 4g). The results indicate that miR-410-3p does indeed have a target binding site on the 3′-UTR of IRS-1 and represses its expression.

### IRS-1 knockdown inhibits adipocyte differentiation and regulation by miR-410-3P

**Fig. 5:**
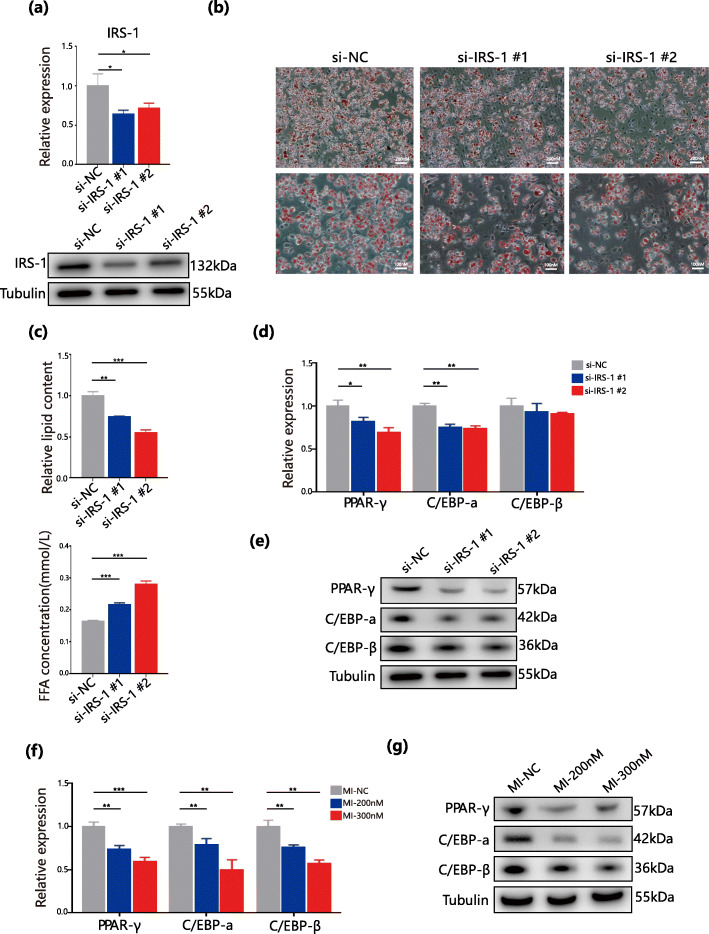
IRS1 knockdown inhibits adipocyte differentiation and is regulated by miR-410-3P. **a**. qPCR analyses and Western Blot analyses of IRS-1 in groups NC and knockdown of IRS-1(#1 and #2). **b**. Oil Red O staining observed with 10X and 20X eyepiece in groups NC and knockdown of IRS-1(#1 and #2). **c**. Quantitative analysis of lipid accumulation and free fatty acids of cell supernatant in groups NC and knockdown of IRS-1(#1 and #2).**d**. qPCR analyses of C/EBP-a, C/EBP-β, and PPAR-γ in groups NC and knockdown of IRS-1(#1 and #2). **e**. Western Blot analyses of C/EBP-a, C/EBP-β and PPAR-γ in groups NC and knockdown of IRS-1(#1 and #2). **f** and **g**. qPCR analyses and Western Blot analyses of C/EBP-a, C/EBP-β and PPAR-γ in NC group and miR-410-3p mimics concentration gradient group. All data are expressed as mean ± SEM. * *P *< 0.05, ** *P*< 0.01, *** *P* < 0.001

To analyse the function of IRS-1 during adipogenesis, two siRNAs were designed for transfection in adipose stem cells, which was followed by induced differentiation. A clear decrease in adipogenesis on day 6 of differentiation was observed in the knockdown group (Fig. 5b and c), and both siRNAs had knockdown efficiencies greater than 60 % (Fig. 5a). Since insulin and IGF-1 play important roles in adipogenesis, research has confirmed that the IRS family has an indispensable function in adipocyte differentiation [26, 27]. This study examined important transcription factors [28, 29] involved in adipogenic differentiation by qPCR, such as CCAAT/enhancer binding protein a (C/EBP-a), CCAAT/enhancer binding protein β (C/EBP-β), and peroxisome proliferator-activated receptor-γ (PPAR-γ) (Fig. 5d). In response to knockdown of IRS-1, C/EBP-a and PPAR-γ, which were clearly downregulated, but not C/EBP-β, consistent results were obtained in the corresponding proteins by western blot (Fig. 5e). The same experiments were performed on the miR-410-3p mimics group and found that C/EBP-a, C/EBP-β and PPAR-γ were all downregulated at the gene expression level (Fig. 5f) with similar downregulation in the corresponding proteins (Fig. 5g). This results, together with previous studies, illustrate that IRS-1 promotes the differentiation of adipocytes and is inversely regulated by miR-410-3p.

### High expression of miR-410-3P in circulating exosomes could be the reason for adipose tissue consumption in cachexia cancer patients

Previous results established that miR-410-3p was indeed an important factor during adipogenesis and was present in very high concentrations within the adipose tissues of CAC patients, which was suggested as one of the underlying mechanisms in cancer patients with irreversible fat wasting. However, the reason for this phenomenon is unknown. Three CAC patients were selected with significant weight loss and obtained their venous blood, which was centrifuged to isolate serum from which exosomes were extracted. Additionally, three noncachexia cancer patients were selected as the normal control (NC) group.

**Fig. 6:**
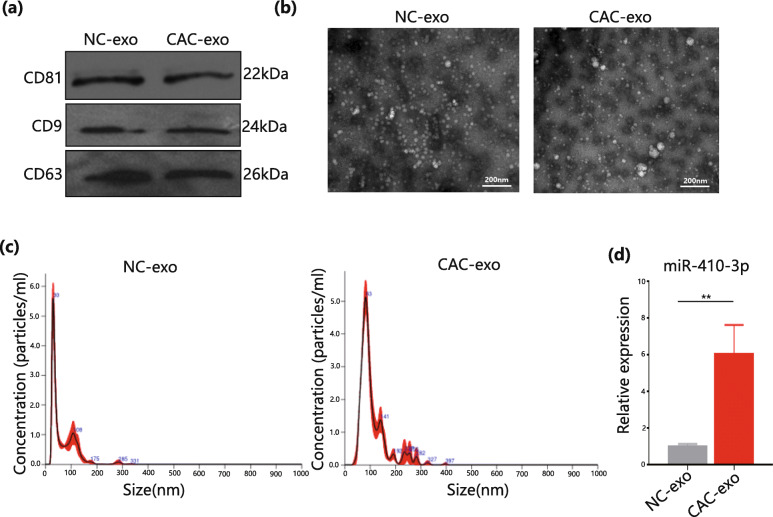
The high expression of miR-410-3P in circulating exosomes could be the reason for adipose tissue consumption in cancer cachexia patients. **a**. Western Blot analyses of common surface markers of serum exosomes in NC group and CAC group. **b**. Transmission electron microscopy (TEM) images under 80 kV and 68,000 times enlargement in NC group and CAC group. **c**. Nanoparticle tracking analysis (NTA) of serum exosomes in NC group and CAC group. **d**. qPCR analyses of miR-410-3p in different groups’ serum exosomes. All data are expressed as mean ± SEM. * *P* < 0.05, ** *P* < 0.01, *** *P* < 0.001

First, to ensure that the extracellular vesicles were dominated by exosomes, this study analysed several common surface markers, including CD9, CD63, and CD81, by western blot and obtained positive results (Fig. 6a). the size and morphology of these vesicles were observed by transmission electron microscopy. Results showed that the vesicles in serum primarily exhibited a double layered vesicular membrane circular structure, which was the conventional morphology of exosomes (Fig. 6b). The vesicles in the NC group were homogeneous and mostly smaller than 50 nm in diameter, whereas those in the CAC group appeared disorganized in shape with numerous large vesicles approximately 100 nm in diameter. In addition, nanoparticle tracking analysis was performed to measure the size and distribution of these vesicles (Fig. 6c). These results also suggested obvious differences in the morphology of vesicles in the serum of two groups, where the diameter of the highest concentration of vesicles was 30 nm in the NC group and 83 nm in the CAC group. qPCR analysis of extracted exosomal miRNAs showed that miR-410-3p exhibited significantly higher expression in exosomes from CAC patients than in exosomes from control subjects (Fig. 6d). As it is difficult to determine the exact origin of exosomes from human serum, the fact that vesicle morphology in CAC patients was markedly different from that in nontumor patients remained evident. The internal environment of humans with tumours may produce unconventional vesicles containing high concentrations of miR-410-3p as a result. This finding, consistent with high concentrations of miR-410-3p within adipose tissues, led us to hypothesize that exosomes might play a critical transduction role in regulating the progression of miR-410-3p in adipose tissues.

## Discussion

CAL is an independent risk factor in cancer cachexia patients, although the current international diagnostic criteria for cachexia do not include adipose tissue loss [30]. To identify possible therapeutic targets for CAC, this study focused on miRNAs, which have not been previously investigated in adipocyte metabolism in CAC. In this study, a differentially expressed miRNA sequencing database of subcutaneous WAT was first used to compare cancer cachexia patients to noncachexia patients, from which 98 differential miRNAs were selected from a primary screening. Several miRNAs have been reported to play an important role in adipocyte metabolism, such as miR-143, miR-431, miR-378, and miR-637 [31–33]. However, as this study needed to identify which miRNA would exert the most significant effect on subcutaneous WAT during the development of CAC, the selection was based on sequencing data of typical patients who were enrolled at the start of this study. From this investigation, it was confirmed that miR-410-3p was highly upregulated in the adipose tissues of CAC in samples from 50 patients, especially when compared to the other nine miRNAs. So miR-410-3p was ultimately selected for follow-up studies. In a comparison study between miR-410-3p expression and the levels of patients’ clinical indicators, it was found that miR-410-3p was indeed associated with cachexia, and the most obvious evidence was its high correlation with weight loss. This study chooses gastric cancer as experiment subjects as the morbidity of cachexia varied in different tumor types yet highest in gastric and pancreatic cancer [34]. In order to reduce bias, 6 patients selected for sequencing were all male and the differentially expressed miRNAs such as miR-410-3p was thus higher.

The function of miR-410-3p was demonstrated in adipocyte metabolism *in vitro* and found that with increasing concentrations of miR-410-3p, adipogenesis was progressively suppressed. However, this study did not observe a clear enhancement of adipogenesis with decreasing concentrations of miR-410-3p. Furthermore, PCR results suggested a low abundance of miR-410-3p in normal adipocytes. This may indicate that miR-410-3p is not a conventional regulator of adipose metabolism; only when the adipocyte microenvironment changes due to the presence of a tumour does the abnormal increase in miR-410-3p affect adipogenesis. Similarly, downregulation of enzymes was observed involved in lipolysis in response to increased miR-410-3p, which may be a negative feedback mechanism to reduce adipogenesis. This relationship was also observed in the inhibitor group.

IRS-1 is a target of miR-410-3p in adipocytes, and according to previous reports, the IRS family plays a crucial role in adipogenic differentiation [35]. This study first demonstrated an inverse relationship between miR-410-3p and IRS-1 and indicated that IRS-1 was indeed a binding target for miR-410-3p via luciferase reporter assay, where vectors for WT and mutant IRS-1 3′-UTR sites were designed. In the knockdown experiment with IRS-1, C/EBP-a and PPAR-γ were clearly downregulated as significant effectors of adipocyte differentiation. This discovery further confirms that insulin and IGF-1 are important factors for adipocyte metabolism in many disease models, including diabetes [36, 37]. Interestingly, it was found that IRS-1 affects the expression of C/EBP-a and PPAR–γ but not CEBP-β, which was consistent with previous research [35]. However, both the CEBP-β gene and protein were downregulated in response to high miR-410-3p concentrations, which was also an important factor in adipogenic differentiation. This suggests the complex effects of miRNA on adipocytes and this study should aim to identify key miRNAs in adipocyte metabolism in CAC. miRNAs could bind to numerous mRNAs to exert abundant regulatory effects. The inhibition of adipogenesis by overexpressing miR-410-3p is a comprehensive phenomenon. This study focuses on the function of IRS-1 in adipose differentiation. There are also other signaling pathways in adipose differentiation which may also be regulated by miR-410-3p.

Recent studies have shown that exosomes play a role in the pathogenesis of various cancers [38]; numerous exosomes are secreted by cancer cells and function in the internal environment [39, 40]. The miRNA can remain stable in these vesicles because the unique physicochemical properties of exosomes protect them from degradation and help them transfer into target cells where they regulate various biological reactions. This study demonstrates that the exosomes obtained from CAC patient sera present distinct morphologies from typical exosomes in tumour-free patients and that a high abundance of miR-410-3p was detected. However, there are multiple differential miRNAs that are highly expressed within exosomes according to previous studies [40, 41].

### Comparisons with other studies and what does the current work add to the existing knowledge

Existing studies considered that miRNAs played an important regulatory role in CAC [41]. Relative regulatory pathways in skeletal muscle had been extensively studied, such as FOXO regulated by miR-206, miR-208 and miR-486 [42, 43]. On the other hand, previous studies had found that miR-378 could enhanced adipocyte lipolysis in CAC. However, the studies focusing on the function of miRNAs in CAL were still few [33]. In this study, miRNAs sequencing in subcutaneous adipose tissues of CAC patients was examined, which could provide data support for subsequent studies about CAL. This study found that miR-410-3p could significantly inhibit adipogenesis and affect the downstream adipose differentiation transcription factor by decreasing the expression of IRS-1, which could become a new predictive marker for CAL and provide a new theory for miRNA regulating adipose loss.

### Study strength and limitations

This study shows that miR-410-3p is an important miRNA in the process of CAC-related adipose tissue loss. miR-410-3p with high abundance in adipocytes may come from serum exosomes and inhibits adipogenic differentiation by downregulation the expression of IRS-1 mRNA. It can be used as an early predictor and provide a new strategy for treatment of cachexia. One limitation of this study is the potential bias of the selection of miRNA based on the sequencing data of typical patients selected in this study.

## Conclusions

Based on results, this study speculates that high concentrations of miR-410-3p in CAC patients’ subcutaneous WAT may be derived from exosomes in serum, and it causes the downregulation of adipocyte differentiation by binding to the noncoding region in IRS-1 mRNA, obstructing the process of lipid accumulation and leading to the development of CAL. The high expression of miR-410-3p in serum exosomes and subcutaneous adipose tissues in cancer patients may become a good marker of cachexia. Early nutritional therapy for patients with high expression could reduce the morbidity of CAC. The results of study strongly suggest that miR-410-3p represents a reliable biological marker for predicting the development of CAL during the early stages of cancer and may potentially be a reversible biological target during the treatment process.

## Supplementary Information

**Additional file 1.**

**Additional file 2: Table S1.** List of PCR primers. **Table S2.** List of miR-410-3P mimics or inhbitors sequences. **Table S3.** List of si-RNA sequences. **Table S4.** Clinical variables of non-cachexia and cachexia patients in miRNA sequencing. **Table S5.** List of miRNAs selected by miRNA sequencing. **Figure S1.** Enrichment analysis of target genes in GO database (screening the top 20 genes in biological process).

**Additional file 3: Table S6. **Target gene of miR-410-3p

## Data Availability

Data are deposited at the Gene Expression Omnibus database with the accession number GSE174128.

## Abbreviations

CAC: Cancer-associated cachexia
CAL: CAC-related adipose tissue loss
WAT: White adipose tissue
3′-UTR: 3′ -noncoding region
HSL: Hormone sensitive lipase
ATL: Adipose triglyceride lipase
CPT1-a: Carnitine palmitoyl transferase-I
IRS-1: Insulin receptor substrate 1
PPAR-γ: Peroxisome proliferator-activated receptor-γ
C/EBP-a: CCAAT/enhancer binding protein a
C/EBP-β: CCAAT/enhancer binding protein β
BMI: Body mass index
FFA: Free fatty acids
miRNAs: Micro RNAs
NTA: Nanoparticle tracking analysis
SEM: Standard error of the mean
FABP4: Fatty acid binding protein 4
AdipoQ: Adiponectin
LPL: Lipoprotein lipase
