# Supplementary figures and images for: miR-410-3P inhibits adipocyte differentiation by targeting IRS-1 in cancer-associated cachexia patients
Source: Lipids Health Dis. 2021 Sep 25;20:115. doi: 10.1186/s12944-021-01530-9 (PMC8465700; doi:10.1186/s12944-021-01530-9)

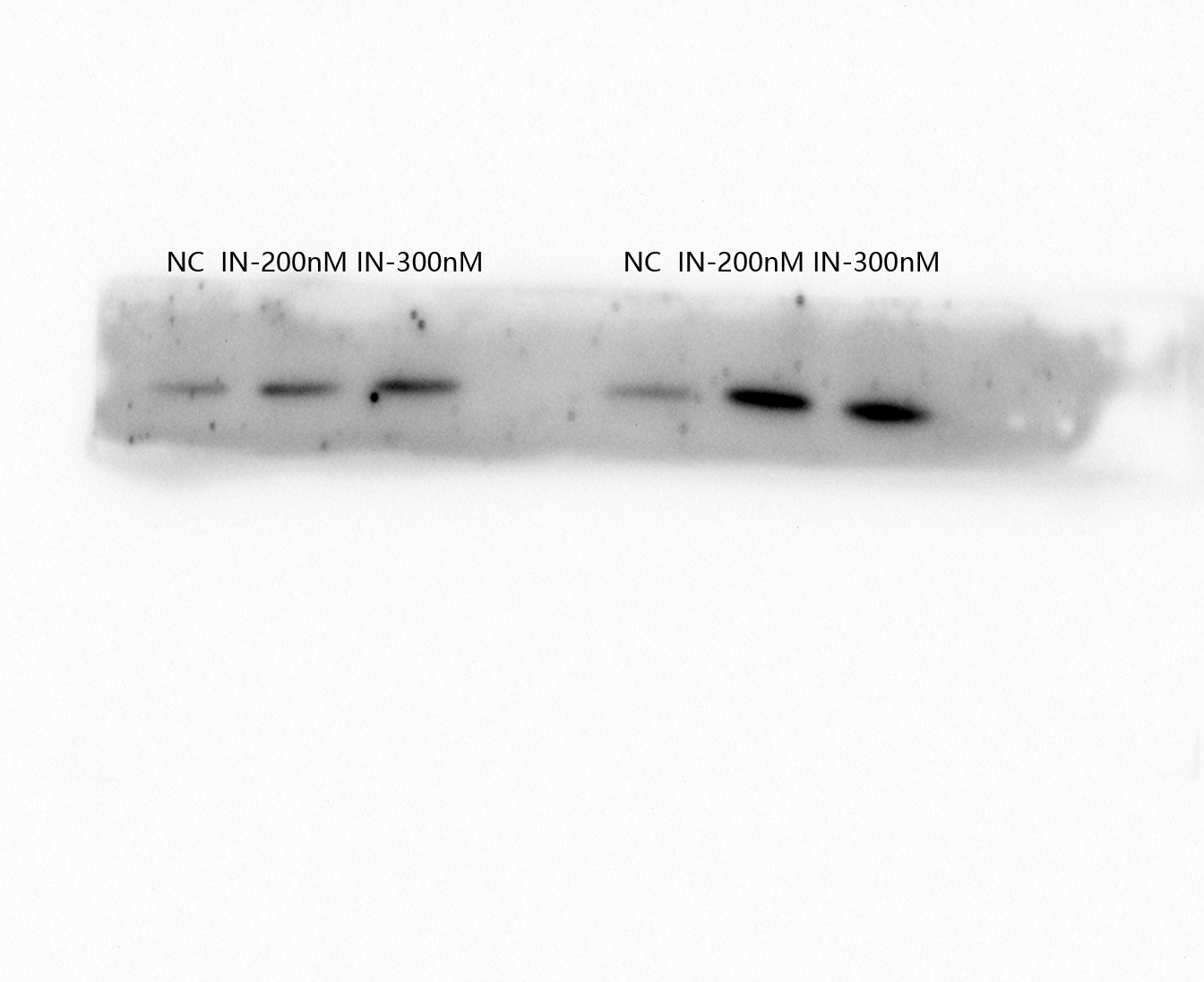

Supplement: Supplementary file 1 — Additional file 1. [file 12944_2021_1530_MOESM1_ESM.tif]
