# Supplementary material for: miR-410-3P inhibits adipocyte differentiation by targeting IRS-1 in cancer-associated cachexia patients
Source: Lipids Health Dis. 2021 Sep 25;20:115. doi: 10.1186/s12944-021-01530-9 (PMC8465700; doi:10.1186/s12944-021-01530-9)
Supplement: Supplementary file 2 — Additional file 2: Table S1. List of PCR primers. Table S2. List of miR-410-3P mimics or inhbitors sequences. Table S3. List of si-RNA sequences. Table S4. Clinical variables of non-cachexia and cachexia patients in miRNA sequencing. Table S5. List of miRNAs selected by miRNA sequencing. Figure S1. Enrichment analysis of target genes in GO database (screening the top 20 genes in biological process). [file 12944_2021_1530_MOESM2_ESM.docx]

**Table S1. List of PCR primers**

| Gene | Forward (5′–3′) | Reverse (5′–3′) |
| --- | --- | --- |
| IRS-1 | CCAGCAGCAGTAGCAGCATCAG | GCTTACCGCCACCACTCTCAAC |
| C/EBP-a | TATAGACATCAGCGCCTACATC | TTCTTGTCCACCGACTTATGAC |
| C/EBP-β | CAAGCTGAGCGACGAGTACA | CAGCTGCTCCACCTTCTTCT |
| LPL | CCTGATGACGCTGATTTTGTAG | CAATGAAGAGATGAATGGAGCG |
| PPAR-γ | GTGCCAGTTTCGATCCGTAGA | GGCCAGCATCGTGTAGATGA |
| FABP4 | CATCCGGTCAGAGAGTACTTTT | TAGGGTTATGATGCTCTTCACC |
| AdipoQ | CCAATGTACCCATTCGCTTTAC | GAAGTAGTAGAGTCCCGGAATG |
| ATGL | CAGAGATGGACTTCGATTCCTT | CAGGTGCTCTAGAATTCGATCT |
| HSL | CTCACAGTTACCATCTCACCTC | GATTTTGCCAGGCTGTTGAGTA |
| CPT1-a | CTACATCACCCCAACCCATATT | GATCCCAGAAGACGAATAGGTT |
| GAPDH | ACTCCACTCACGGCAAATTC | TCTCCATGGTGGTGAAGACA |

**Table S2. List of miR-410-3P mimics or inhbitors sequences**

| miR-410-3P | Sequence(5'-3') |
| --- | --- |
| mimics | AAUAUAACACAGAUGGCCUGU |
|  | AGGCCAUCUGUGUUAUAUUUU |
| inhibitors | ACAGGCCAUCUGUGUUAUAUU |

**Table S3. List of si-RNA sequences**

| siRNA | Sequence(5'-3') |
| --- | --- |
| si-NC | UUCUCCGAACGUGUCACGUTT |
| si-IRS-1#1 | GGCUUCUAUUGAGGAAUAUTT |
| si-IRS-1#2 | GAGGAGCUGAGCAAUUAUATT |

**Table S4. Clinical variables of non-cachexia and cachexia patients in miRNA sequencing**

| Features | Cachexia(n=3) | Non-cachexia(n=3) | t | p |
| --- | --- | --- | --- | --- |
| Gender | Only male | |  |  |
| Age(years) | 64.33±4.92 | 57.67±12.28 | 0.783 | 0.516 |
| Weight(kg） | 63±3.26 | 69±3.74 | 2.166 | 0.163 |
| Body weight loss(%) | 14.63±5.95 | 0±0 | 6.155 | 0.025 |
| BMI(kg/m2) | 21.12±0.9 | 24.82±0.72 | 9.7 | 0.01 |
| TNM stage | Only III stage | Only II stage |  |  |
| IL-6（pg/ml） | 5.47±2.32 | 2.3±0.14 | 2.544 | 0.126 |
| TNF-a（pg/ml） | 8.63±3.48 | 7.7±2.71 | 0.319 | 0.78 |
| Total protein (g/L) | 59.33±2.05 | 62.67±2.05 | 2.026 | 0.18 |
| Albumin(g/L) | 34.33±2.87 | 40±0.82 | 4.095 | 0.055 |

**Table S5. List of miRNAs selected by miRNA sequencing**

| miRNA ID | Sequence (5’→ 3’) |
| --- | --- |
| hsa-miR-122-5p | TGGAGTGTGACAATGGTGTTTG |
| hsa-miR-1-3p | TGGAATGTAAAGAAGTATGTAT |
| hsa-miR-337-3p | CTCCTATATGATGCCTTTCTTC |
| hsa-miR-323a-3p | CACATTACACGGTCGACCTCT |
| hsa-miR-329-3p | AACACACCTGGTTAACCTCTTT |
| hsa-miR-485-3p | GTCATACACGGCTCTCCTCTCT |
| hsa-miR-431-5p | TGTCTTGCAGGCCGTCATGCA |
| hsa-miR-411-3p | TATGTAACACGGTCCACTAACC |
| hsa-miR-495-3p | AAACAAACATGGTGCACTTCTT |
| hsa-miR-548ay-5p | AAAAGTAATTGTGGTTTTTGC |
| hsa-miR-410-3p | AATATAACACAGATGGCCTGT |


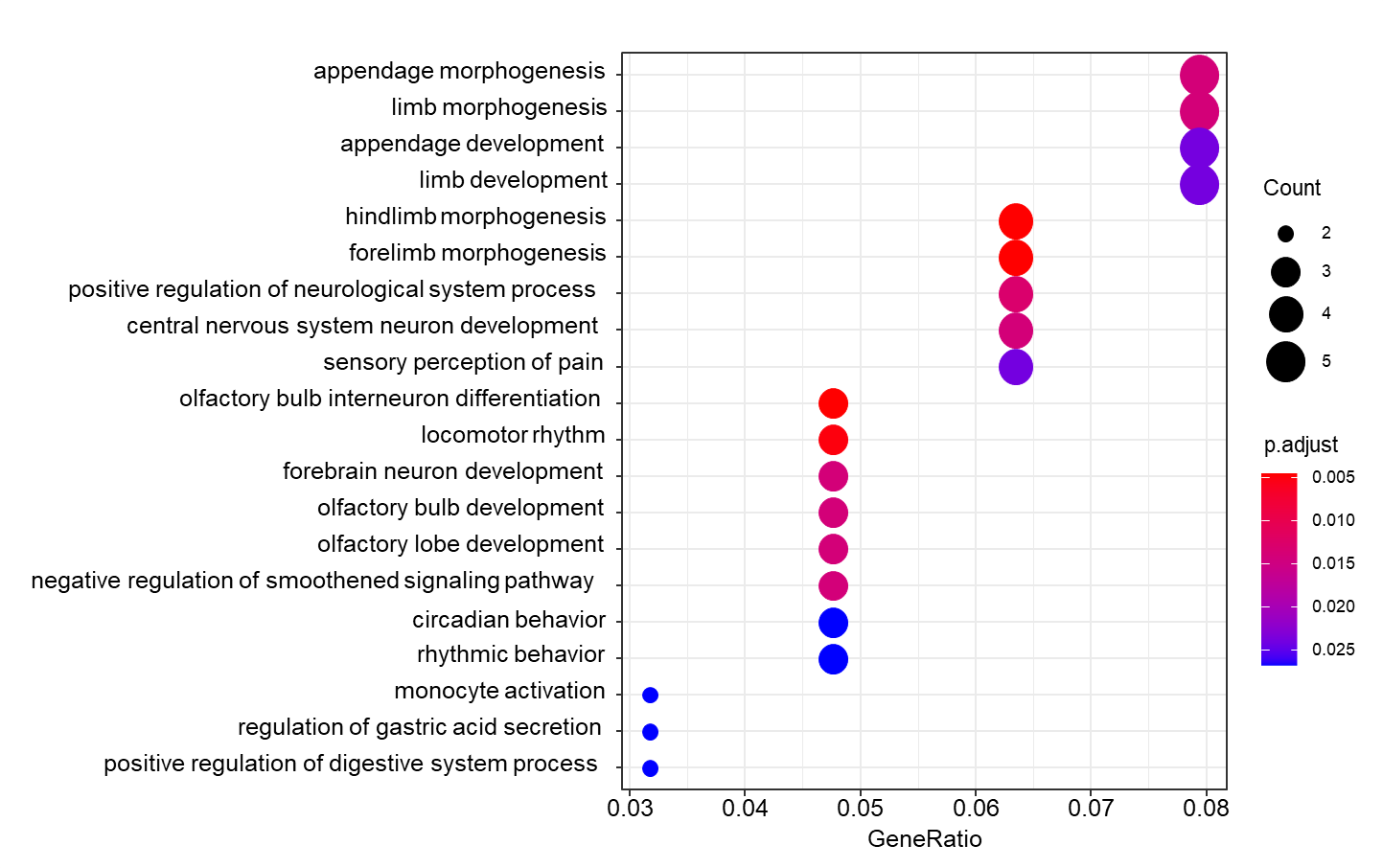
**Figure S1. Enrichment analysis of target genes in GO database (screening the top 20 genes in biological process)**
